# The Relationship between Genus Richness and Geographic Area in Late Cretaceous Marine Biotas: Epicontinental Sea versus Open-Ocean-Facing Settings

**DOI:** 10.1371/journal.pone.0040472

**Published:** 2012-08-03

**Authors:** Anne J. Lagomarcino, Arnold I. Miller

**Affiliations:** Department of Geology, University of Cincinnati, Cincinnati, Ohio, United States of America; Monash University, Australia

## Abstract

For present-day biotas, close relationships have been documented between the number of species in a given region and the area of the region. To date, however, there have been only limited studies of these relationships in the geologic record, particularly for ancient marine biotas. The recent development of large-scale marine paleontological databases, in conjunction with enhanced geographical mapping tools, now allow for their investigation. At the same time, there has been renewed interest in comparing the environmental and paleobiological properties of two broad-scale marine settings: *epicontinental seas*, broad expanses of shallow water covering continental areas, and *open-ocean-facing settings*, shallow shelves and coastlines that rim ocean basins. Recent studies indicate that spatial distributions of taxa and the kinetics of taxon origination and extinction may have differed in these two settings. Against this backdrop, we analyze regional Genus-Area Relationships (GARs) of Late Cretaceous marine invertebrates in epicontinental sea and open-ocean settings using data from the Paleobiology Database. We present a new method for assessing GARs that is particularly appropriate for fossil data when the geographic distribution of these data is patchy and uneven. Results demonstrate clear relationships between genus richness and area for regions worldwide, but indicate that as area increases, genus richness increases more per unit area in epicontinental seas than in open-ocean settings. This difference implies a greater degree of compositional heterogeneity as a function of geographic area in epicontinental sea settings, a finding that is consistent with the emerging understanding of physical differences in the nature of water masses between the two marine settings.

## Introduction

One of the most fundamental aspects of biodiversity is the relationship between species richness and the size of habitable area. Known as the *Species-Area Relationship (SAR)*, this property, and how its parameters vary among regions, has been documented for many present-day taxa, and conservation biologists have recognized its predictive power for estimating biodiversity both in continental regions and on islands of various sizes [1]–[6].

It is well understood that in the present day and throughout the history of life many factors besides the amount of habitable area have likely also mediated diversity, including those that govern the *environmental heterogeneity* of a given region. By understanding the effects of these mitigating factors on the number of species expected in a given area, it should be possible to understand why regions of comparable areal extent might harbor different numbers of species.

Against this backdrop, recent research has highlighted an important secular transition among marine settings through the Phanerozoic eon: a decline in the extent of *epicontinental seas*, the broad, shallow expanses that once covered major continental areas; and a concomitant increase in the proportion of the record characterized by *shallow open-ocean facing settings*, such as the continental shelves that rim ocean basins [7]–[9]. Differences between the two regimes in physical properties such as the nature of water circulation, the propagation of physical disturbances, and the steepness of onshore-offshore gradients, may have been associated with biological differences in origination and extinction rates, as well as the degree of isolation or regionalization of water masses and biotas [10]–[13], [8], [9], [14], [15]. These factors, in turn, suggest that the two systems may exhibit different quantitative relationships between taxonomic richness and area.

While most present-day studies of the relationship between area and diversity have been conducted at the species level, one could just as easily look at these relationships at higher taxonomic levels, and, because of concerns about data quality, all analyses in this paper were conducted at the genus level, which has been the level of choice for a broad suite of investigations of ancient marine diversity conducted over the past two decades. When referring to studies conducted at the species-level we will use the term *Species-Area Relationships (SARs)*, whereas when referring to analyses within this study we will use the term *Genus-Area Relationships (GARs).*

The purpose of this paper is threefold: 1) to establish the viability of investigating GARs at regional scales for marine biotas preserved in the fossil record; 2) to introduce a new protocol for analyzing GARs that may be particularly appropriate for the spatial distribution of data available in the fossil record; and 3) to compare and contrast the nature of marine invertebrate GARs in epicontinental seas versus shallow-ocean facing settings through the Late Cretaceous, a Phanerozoic interval when both settings are well represented in the record.

To date, there have been only limited studies of taxon-area relationships in the fossil record, with most focused on terrestrial mammals [16]–[19]. Little is known about these relationships for fossil marine invertebrates, whose more extensive records have long been the basis of most investigations of long-term diversity trends. Historically, the data required for these investigations have not been readily available, but the recent development of large-scale paleontological databases that contain extensive marine data (e.g. the Paleobiology Database; http://www.paleodb.org) in conjunction with the development of enhanced geographical and paleogeographical mapping tools, now facilitates investigation of taxon-area relationships in the marine geological record.

Our analyses suggest that GARs differ between epicontinental seas and shallow open-ocean facing settings during the Late Cretaceous. As illustrated below, the slope of the relationship is generally higher for epicontinental seas, implying that these settings supported greater numbers of genera per unit area. Additionally, GARs varied temporally within each setting. Specifically the slope was significantly higher during a Cretaceous interval that coincides, based on other recent analyses [20], with peak global diversity during the Late Cretaceous.

**Figure 1:**
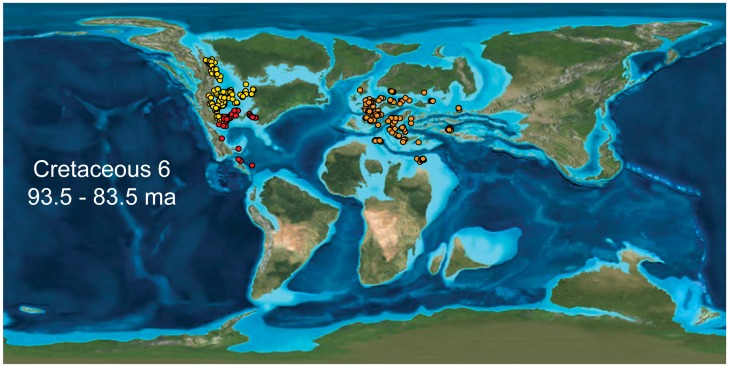
PaleoDB collections from the Cretaceous 6 bin. The three regions examined: European Epicontinental Sea (orange), North American Cretaceous Seaway (yellow), and Gulf Coast (red). Map: Ron Blakey, NAU Geology [21].

## Methods

### Data

The data used here is the Late Cretaceous portion of a dataset analyzed by Miller and Foote [9] in their investigation of origination and extinction rates in epicontinental seas versus open-ocean settings in the Permian through Cretaceous periods. Stage-to-stage global occurrences, including paleolatitudinal and paleolongitudinal coordinates for all marine metazoan genera, excluding tetrapods, were downloaded from The Paleobiology Database (PaleoDB) on July 17, 2009, and were filtered for problematic taxonomic occurrence. Collections were designated as either *open-ocean-facing* or *epicontinental* based their proximities to deep ocean settings as depicted on global paleogeographic maps [21]. For each stratigraphic interval in the Late Cretaceous, a grid of 5° latitude by 5° longitude cells was first superimposed on the paleogeographic map, and then cells isolated from deep oceanic areas by at least one 5° by 5° cell were designated as *epicontinental*, whereas those within one cell of open-ocean areas were designated as *open-ocean-facing* (see the supplementary online material for Miller and Foote [9] for additional details). Analyses in the present study were limited to one open-ocean facing setting, the North American Gulf Coast, and two well-known Late Cretaceous epicontinental seas, the broad, shallow region extending over much of present-day Europe, the Mediterranean, and North Africa, and the North American Cretaceous Interior Seaway (Fig. 1). Given the areal breadth of the first epicontinental region and the possibility that it encompasses more than one biogeographic realm [22], some analyses were also conducted after parsing the region into separate northern and southern portions.

**Figure 2:**
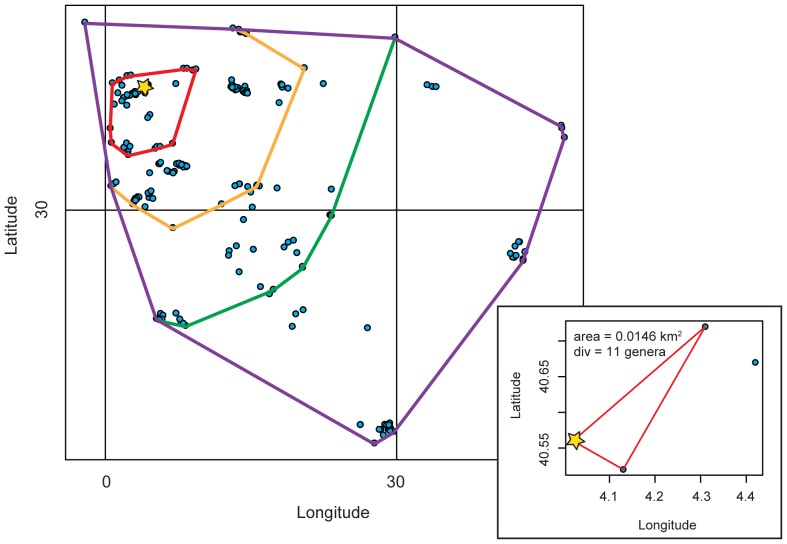
Spatial protocol used in this study to build genus-area curves from fossil data. Blue dots represent fossil collections in the PaleoDB of marine invertebrates during the Cretaceous 6 time bin from the European Epicontinental sea. Inset: One collection was randomly chosen as a start point (yellow star); a convex hull was circumscribed around the start collection and the next two closest collections. Main figure: Each collection was added individually and a new convex hull was circumscribed around collections in order to calculate area. The rainbow lines represent a few convex hulls from this iteration. This process was then repeated using each collection as a start point.

**Figure 3:**
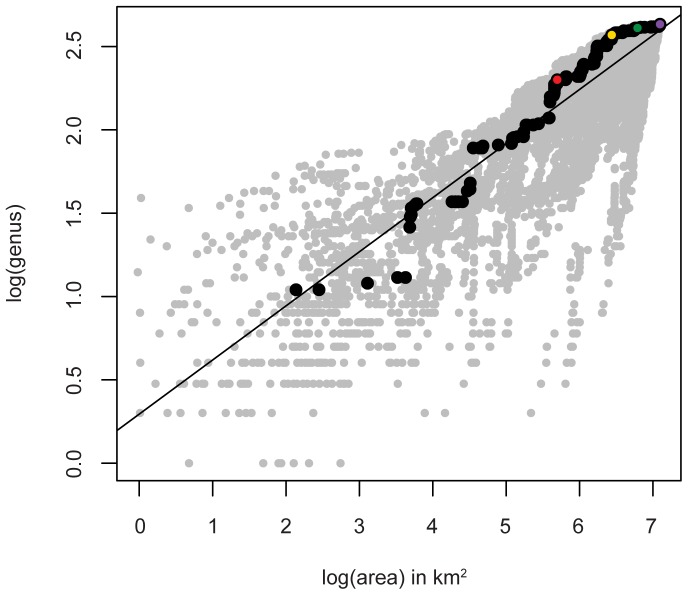
Example of multiple genus-area relationship (**GAR**) **analyses plotted together.** Gray dots represent all iterations regionally; black dots represent the single iteration illustrated in Fig. 2. The colored dots correspond to the hulls pictured in Fig. 2. Data are from the European Epicontinental Sea during the Cretaceous 6 time bin.

Following the convention of Alroy et al. [20] and Miller et al. [23], the Late Cretaceous was parsed into four temporal bins of roughly equal duration (the “standard” PaleoDB ∼10 my bins), referred to here and in other studies as *Cretaceous 5* through *Cretaceous 8*. Genus-area analyses were conducted on the data contained within each of these bins. For cross-comparisons with this approach, analyses were also conducted at the stage level, because of suggestions that, despite inequalities in stage length, stages contain more temporally homogenous biotas. The stages comprising Cretaceous 5 through 8 and analyzed here include the Cenomanian (Cretaceous 5), Turonian (Cretaceous 6), Coniacian (Cretaceous 6), Santonian (Cretaceous 6), Campanian (Cretaceous 7), and Maastrichtian (Cretaceous 8). Note that Cretaceous 6 is the only PaleoDB bin in the study intervals that contains more than one stage.

### Taxon-area equations

In this study we implemented a widely used depiction of the species-area relationship, the power law function:


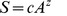
(1)

where *S* is the number of species, *A* is the area, and *c* and *z* are empirically derived constants that express the slope and intercept in the logarithmic transformation:


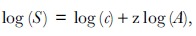
(2)

**Figure 4:**
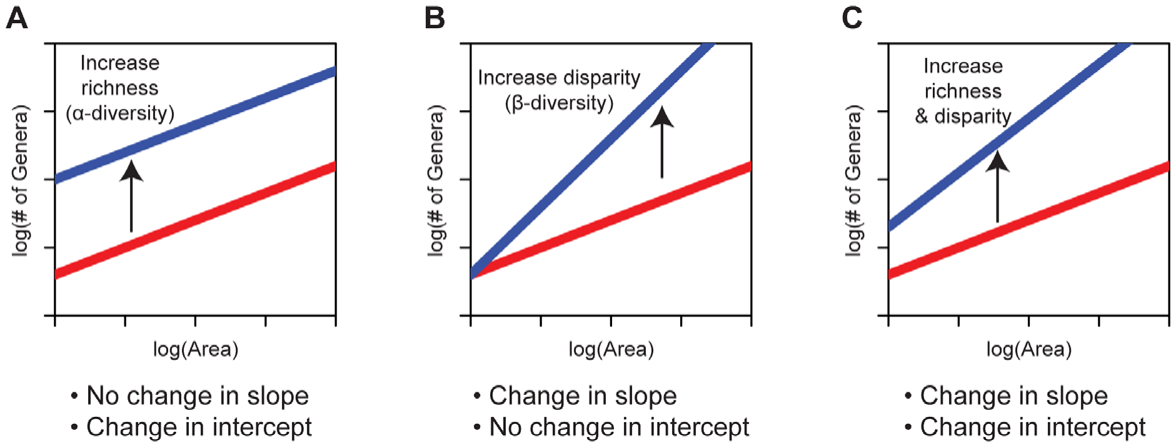
Changes to the genus-area relationship best-fit line due to changes in alpha and beta diversity. (A) Alpha-diversity is higher in the blue region; beta-diversity is identical. (B) Alpha-diversity is identical in the two regions; beta-diversity is higher in the blue region. (C) Both alpha and beta-diversity are higher in the blue region.

**Figure 5:**
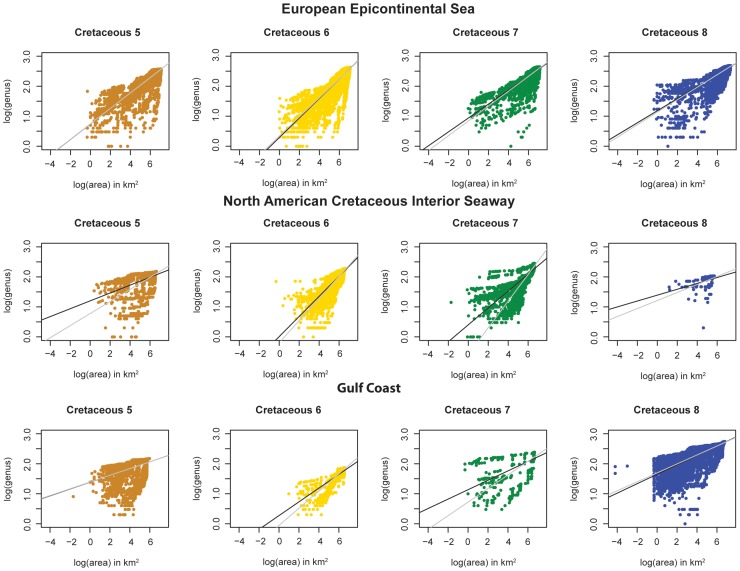
Genus-Area Results. Genus-area (GAR) plots of the European Epicontinental Sea, the North American Cretaceous Interior Seaway and the Gulf Coast, an open-ocean-facing setting. Cretaceous 5–8 represent four ∼11 million year time bins spanning the Late Cretaceous (see text for discussion of time scale). Ordinary least-squares (black) and generalized least-squares (gray) linear regressions are also plotted. All slopes are highly significant, and each is significantly different from all others. Note: when it appears that only one regression line is present this is because the two lines coincide.

**Table 1:** P-values for tests of significant differences between individual genus-area linear regression slopes.

	*E5*										
*E6*	**<0.0001**	*E6*									
*E7*	**<0.0001**	**<0.0001**	*E7*								
*E8*	**<0.0001**	**<0.0001**	**<0.0001**	*E8*							
*N5*	**<0.0001**	**<0.0001**	**<0.0001**	**<0.0001**	*N5*						
*N6*	**<0.0001**	**<0.0001**	**<0.0001**	**<0.0001**	**<0.0001**	*N6*					
*N7*	**<0.0001**	**<0.0001**	**<0.0001**	**<0.0001**	**<0.0001**	**<0.0001**	*N7*				
*N8*	**<0.0001**	**<0.0001**	**<0.0001**	**<0.0001**	0.1268	**<0.0001**	**<0.0001**	*N8*			
*G5*	**<0.0001**	**<0.0001**	**<0.0001**	**<0.0001**	**0.00008**	**<0.0001**	**<0.0001**	0.4401	*G5*		
*G6*	**<0.0001**	**<0.0001**	0.6562	**<0.0001**	**<0.0001**	**<0.0001**	**<0.0001**	**<0.0001**	**<0.0001**	*G6*	
*G7*	**<0.0001**	**<0.0001**	**<0.0001**	**<0.0001**	**0.00002**	**<0.0001**	**<0.0001**	0.015	**<0.0001**	**<0.0001**	*G7*
*G8*	**<0.0001**	**<0.0001**	**<0.0001**	**<0.0001**	**<0.0001**	**<0.0001**	**<0.0001**	**<0.0001**	**<0.0001**	**<0.0001**	0.7807

**Figure 6:**
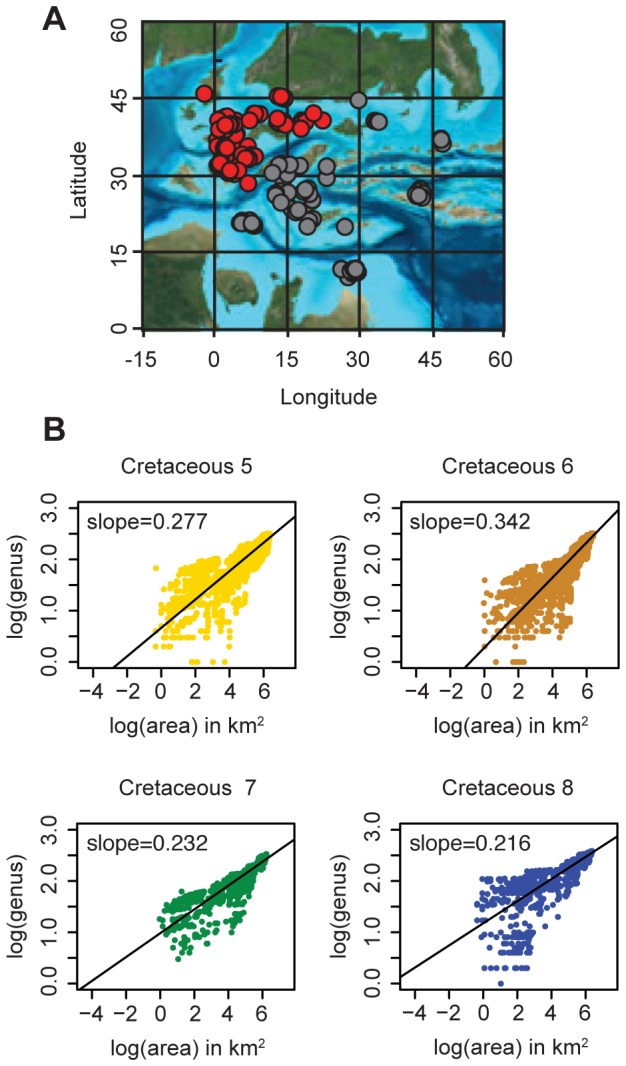
European Epicontinental Sea PaleoDB Collections. (A) Paleogeographic map of Europe (105Ma). Gray dots represent all PaleoDB collections from the European Epicontinental Sea used in full analyses; red dots represent collections used as a subset for the European Epicontinental Sea analysis. Map: Ron Blakey, NAU Geology [21]. (B) Genus-Area plots with least-squares linear regression of subseted European Data.

Comparison of regression lines was performed using ANCOVA, with a Bonferroni corrected alpha value of 0.0008. Regressions (shown in Fig. 6) that are significantly different are bolded. Each regression is labeled by PaleoDB time bin, 5–8, and with an “E” for the European Epicontinental Sea, an “N” for the North American Cretaceous Interior Seaway or a “G” for the Gulf Coast.

[1], [24], [4], [25], [26]. We chose to use the logarithmic transformation of the power law function, substituting genera for species, for three reasons: 1) it is the most commonly used mathematical representation of species-area relationships in the literature, and therefore allows for easy comparison to previous studies; 2) it has been suggested that the power law is the most appropriate for analyses spanning intermediate to large sampling areas [27], [26]; and 3) for a nested-squares spatial protocol (the spatial sampling scheme closest to that used in this study; see *Spatial Sampling Protocol* section) the power function is the most robust of two-parameter species-area models [28].

### Spatial sampling protocol

A variety of spatial protocols have been designed to sample a region for assessment of taxon-area relationships for present-day taxa and settings [25]. However, because of the non-uniform spatial distribution of fossil collections, as well as differences in the number of occurrences per collection in the PaleoDB and, likely, any other database, none of the currently established methods seemed appropriate for the present study. From a geographic perspective, fossil occurrences are often distributed haphazardly because of the sporadic nature of rock and fossil exposures, as well as geographic variation in researcher interest and effort. To avoid idiosyncratic changes in the slope of the GAR associated with the patchy addition of large groups of collections or occurrences at some point when beginning with a single, perhaps arbitrarily chosen, starting point, a new protocol was developed for this study that is broadly related to a nested-plots approach [25], but with an added dimension to the protocol, as described below.

**Table 2:** Slope and intercept estimates for ordinary least squares (OLS) and generalized least-squares (GLS) linear regression.

	OLS slope	GLS slope	OLS intercept	GLS intercept
***European Epicontinental Sea***				
Cretaceous 5	0.258	0.257	0.743	0.747
Cretaceous 6	0.325	0.317	0.294	0.344
Cretaceous 7	0.231	0.247	0.943	0.847
Cretaceous 8	0.201	0.208	1.189	1.139
***North American Cretaceous Interior Seaway***				
Cretaceous 5	0.131	0.201	1.2	0.778
Cretaceous 6	0.335	0.369	0.012	−0.194
Cretaceous 7	0.282	0.444	0.402	−0.585
Cretaceous 8	0.098	0.134	1.391	1.212
***Gulf Coast***				
Cretaceous 5	0.113	0.112	1.385	1.389
Cretaceous 6	0.229	0.287	0.272	−0.057
Cretaceous 7	0.157	0.226	1.135	0.722
Cretaceous 8	0.157	0.149	1.648	1.695

First, instead of increasing each plot by a predetermined amount, collections were added individually and sequentially, based on spatial proximity from the location of an initial, starting collection. We chose this initial collection at random (Fig. 2); then, based on great circle distance, we selected the two collections closest to this point (see inset Fig. 2). The genus richness of the three combined collections was determined, and the area of the smallest polygon that could be circumscribed to include the collections (a triangle in this first case) was determined using a convex-hull calculation [29]. This method was repeated, adding collections one-by-one, until all collections in the region were included. The colored polygons in Figure 2 represent four incremental examples from the much larger set of polygons for which area and richness were calculated in the case of the European Epicontinental Sea for the Cretaceous 6 time bin; the largest (purple) polygon represents the final, largest area, encompassing all of the collections.

Because of unevenness in coverage and the possibility that the chosen starting point could, itself, greatly affect the shape of the genus-area curve, multiple analyses were conducted for each region in which *every* collection served as the starting point for one analysis, with the results for all analyses superimposed as points on a single scatter plot (Figure 3). Ordinary least squares linear regression (OLS) was conducted on the total scatter of points from this pooled set of analyses in a given region. The slope of the regression is the estimate of the regional rate at which genus richness accumulates as area increases. The slopes of the least squares linear regressions were compared using ANCOVA; a Bonferroni corrected alpha value of 0.0008 was used. Because of the heteroscedastic nature of our data generalized least squares regression (GLS) was also conducted. The GLS solution presented here was conducted assuming uncorrelated errors and an exponential heteroscedasticity structure; other structures were tested and yielded nearly identical results. To diagnose any possible systematic changes in the genus-area relationship as area increases, LOESS regression was also conducted.

**Figure 7:**
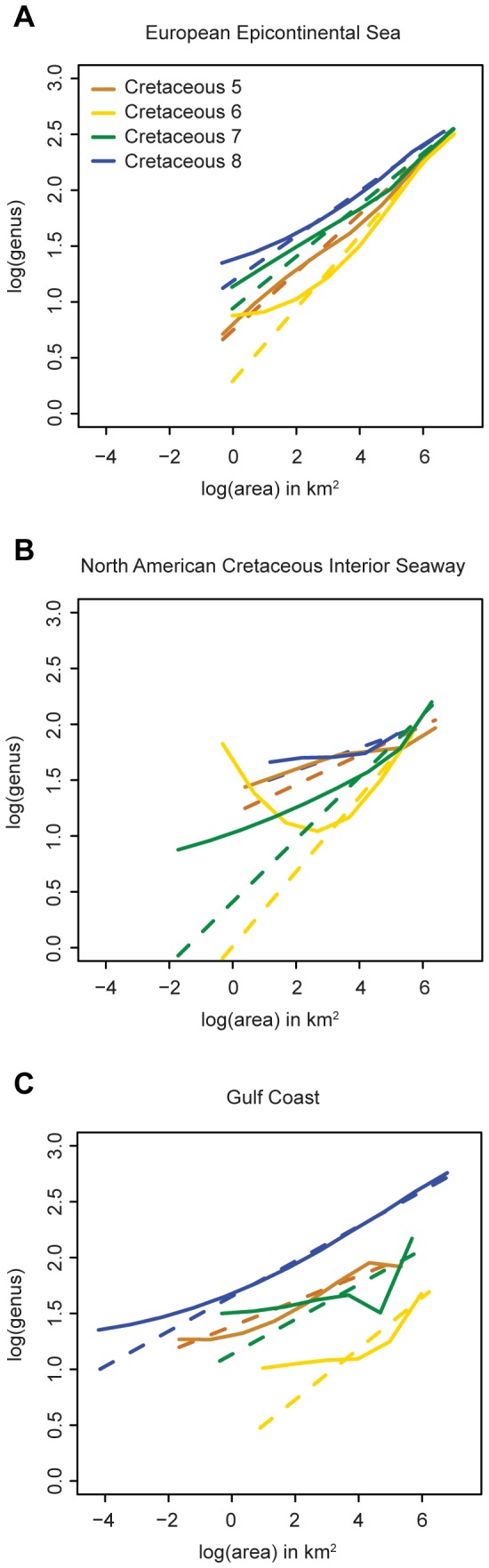
A comparison between least squares linear regression and LOESS regression. Least squares linear regression is displayed with dashed lines; LOESS by solid lines for (A) the European Epicontiental sea, (B) the North American Cretaceous interior seaway, and (C) the Gulf coast. LOESS was conducted with an alpha (smoothing parameter) of 0.4.

While this method is somewhat unconventional compared to previous taxon-area analyses, we suggest that it appropriately addresses the uneven spatial distribution inherent in most fossil data sets, by combining in a single analysis every possible starting point within a region in question. The scatter reflects variations in estimates of genus richness for regions of various sizes associated with variations in the starting points.

All analyses and graphics were produced using programs written in the R programming language [30].

**Figure 8:**
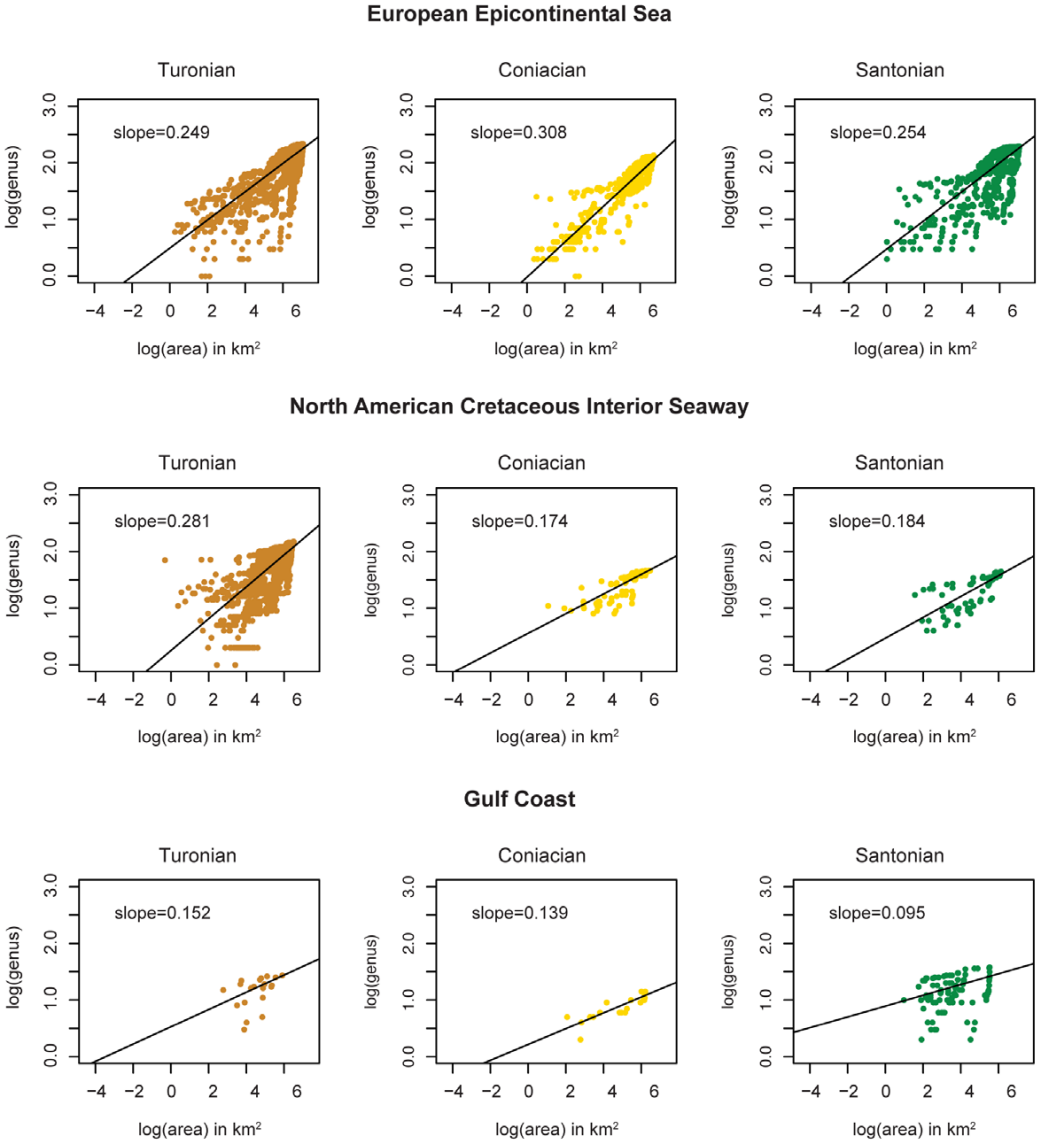
Stage level genus-area plots. Shown here are the European Epicontinental Sea, the North American Cretaceous Interior Seaway and the Gulf Coast for the Cretaceous 6 time bin, which includes stages: Turonian, Coniacian and Santonian. Time bins Cretaceous 5, 7 and 8 each span only 1 stage: Cenomanian, Campanian and Maastrichtian respectively.

**Figure 9:**
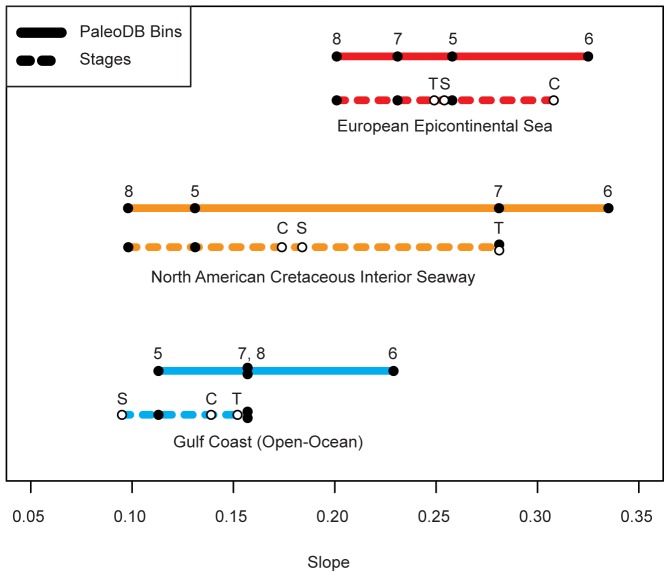
Range of least-squares linear regression slopes for each region. Black dots represent actual slope values, while the colored lines represent the range in values for a given region. Solid lines represent values for analyses using PaleoDB temporal bins (Cretaceous 5–8); dotted lines represent slope values using stage divisions. White dots represent the stages within the Cretaceous 6 PaleoDB time bin: Turonian (T), Coniacian (C) and Santonian (S).

### Alpha and beta diversity

Importantly, within habitat (alpha) and between-habitat (beta) diversity values that are characteristic of a given region may fundamentally affect the slopes and intercepts of genus-area curves derived for the region. *Alpha diversity* here refers to the diversity at a single locality or of an individual community; *beta diversity* refers to the number of unique taxa between communities or localities. The slope of the power law function (equation 1) has often been used as a measure of beta diversity, whereas the intercept has been taken as a measure of alpha diversity [31], [3], [4], [32], [33].

Figure 4 presents comparative schematic representations of likely changes in regression estimates of regional genus-area curves for two hypothetical regions (shown in blue and red), in association with variations in alpha and beta diversity. If the two regions have similar beta diversity but different alpha diversity, the best-fit lines should have similar slopes (Fig. 4A), because they would be expected to add diversity at a similar rate per unit addition of area. However, because individual assemblages would be more taxon-rich in the region with higher alpha-diversity, the best-fit line for this region should have a higher y-intercept than that for the region with lower alpha diversity (Fig. 4A). If, instead, both regions have similar alpha diversity but different beta diversity, the y-intercept of the two best-fit lines would be similar because taxon richness would be similar for individual assemblages, but the slope would be steeper for the region with higher beta diversity because it would be expected to add taxa at a higher rate as area is added (Fig. 4B). Finally, Figure 4C illustrates a scenario in which both alpha and beta diversities differ, and, therefore, both the slope and intercept of the regression lines differ.

## Results and Discussion

Figure 5 illustrates the relationship between genus richness and geographic area during the Late Cretaceous for the three study regions. Each regression is significant, *i.e.* genus diversity and geographic area are significantly correlated, and the slope of each regression is significantly different from those of all other illustrated analyses, with a few exceptions (see Table 1 for p-values). Two patterns stand out immediately:

1. For a given time interval, the slopes of the two epicontinental sea regions are, with one exception, greater than that of the coeval open-ocean setting. The only exception is the comparison in Cretaceous 8 (the Maastrichtian) between the North American Cretaceous Interior Seaway and the Gulf Coast, but this may relate to the relative scarcity of data for the North American Cretaceous Interior Seaway during that interval. These results are robust to the OLS and GLS treatments of the data.
2. OLS indicates that the Cretaceous 6 temporal bin has a significantly higher slope than the other temporal bins for all three regions. With GLS, however, this pattern holds only for the European Epicontinental Sea and the Gulf Coast. Regression estimates are shown in Table 2.

The higher slopes in the two epicontinental sea examples indicate that genus richness increases at a greater rate as area is added than in the open-ocean setting. That said, the data from the European Epicontinental Sea actually might have spanned two ancient provinces, the Southern European Bioprovince and the Tethys Bioprovince [22], covering about two times the geographic area of the other regions. Analyses were therefore also conducted on a subset of that dataset including only the Southern European Bioprovince (Fig. 6A). When this was done, the slopes of the linear regressions for the subsets were nearly the same as those for the larger, coeval datasets (Fig. 6B). The higher slope of the genus-area relationship in epicontinental sea settings, therefore, appears to relate to real physical and biological attributes of these settings, and not differences in areal extent of coverage or the crossing of provincial boundaries.

In addition to OLS and GLS, LOESS regression was also conducted (Fig. 7) to diagnose changes, if any, in slope, possibly associated with crossing into different environments as areal coverage was increased [4], [25]. The LOESS regressions fall consistently above the least-square linear regressions at small areas. For larger areas (>100–1000 km^2^), LOESS regressions generally converge on the least squares linear regressions, with the exception of Cretaceous 7 of the Gulf Coast, which takes an unusual downward excursion. LOESS regression for the North American Cretaceous Interior Seaway during the Cretaceous 6 interval deviates significantly from least squares linear regression; this interval also exhibits the greatest difference between OLS and GLS slope estimates. This anomaly relates to one data point (see Fig. 5) that exhibits high richness at a very small areal extent, with no other data points. However, this LOESS curve still follows the same general pattern as all of the other LOESS regressions.

It is unsurprising that least squares linear regression underestimates the number of genera expected for small areas because at a small-scale the addition of a single collection will add more unique genera than later collections (this is why sampling curves are steeper at the beginning). While least squares linear regression may not be a good fit for GARs at small geographic ranges, it does seem to capture the larger, regional signal.

As indicated by the schematic illustrations presented and discussed earlier (Fig. 4), the difference in least squares slopes between the epicontinental-sea settings and the open-ocean Gulf Coast may be indicative of a greater spatial disparity or beta-diversity in epicontinental seas. In some ways, this result seems counterintuitive, because one might expect to observe steeper environmental gradients and, therefore, greater beta diversity on open-ocean coastlines, where there may have been more pronounced depth gradients. There is growing evidence, however, that, owing to very sluggish circulation, water masses in epicontinental seas may become highly localized, with concomitant localization of biotas [10]–[12], [13], [8], [9], [14], [15]. This would serve to steepen the slopes of the GARs for epicontinental seas. On a global scale, in fact, there is evidence that during the Late Cretaceous, the geographic ranges of marine genera in epicontinental settings were generally significantly smaller than those of their open-ocean counterparts (see Fig. 1C in Miller and Foote [9]). Conversely, increased dispersal has been shown to lower the slopes of SARs with individual assemblages becoming more similar to one another (Drakare et al. [34] and references therein).

The steeper slopes of GARs in all regions for the Cretaceous 6 relative to the other three (Fig. 5) coincide with the interval of peak Phanerozoic diversity identified in Alroy et al.'s [20] sampling-standardized global marine diversity curve. It is possible, therefore, that increased beta diversity, as indicated by the increased slopes in Cretaceous 6, contributed to the unusually high level of global diversity observed during that interval. Alternatively, because it includes three global (bio) stratigraphic stages despite encompassing roughly the same amount of time as the other three temporal bins, which each include just a single stage, Cretaceous 6 may have been a time of unusually rapid evolutionary turnover. This heightened turnover, if it occurred, may have artifactually enhanced the apparent geographic disparity observed during this interval. To investigate this possibility, the data for Cretaceous 6 were also parsed and analyzed at the stage level (Figs. 8 and 9). Relatively high GAR slopes were maintained from stage to stage for the European Epicontinental Sea and the North American Cretaceous Interior Seaway, but relative stage-level slopes for the Gulf Coast were not as consistently high. This may reflect a real difference between the way that diversity was partitioned in Cretaceous 6 epicontinental seas versus open-ocean settings or, may instead, reflect a relative paucity of data for individual Cretaceous 6 stages along the Gulf Coast (Fig. 8). However, stage-level slopes are not nearly as high for individual stages as they are for the aggregate Cretaceous 6 interval, and are more in line with observed slopes within their respective regions. This, in turn, suggests that the comparatively elevated slopes for Cretaceous 6 at least partly reflect stage-to-stage taxonomic turnover through the interval.

While beta-diversity appears higher in epicontinental seas, alpha-diversity, estimated by the intercepts of our regressions lines, may in fact be higher in the Gulf Coast for all time intervals except Cretaceous 6 (or if looking at GLS estimates Cretaceous 6 & 8), when the intercepts of the European Epicontinental Sea and the Gulf Coast are nearly identical (Table 2). It therefore appears that, in contrast to beta diversity, there may be a trend of lower alpha diversity in the epicontinental-sea settings. These results are consistent with Bambach's observation that alpha-diversity was higher in open-ocean settings than in epicontinental settings during the Paleozoic era, and furthermore indicates that this pattern continues into the Mesozoic, however at this point the results are equivocal [35].

The method presented here for the analysis of taxon-area relationships is particularly appropriate for fossil data, when the geographic distribution of these data is patchy and uneven. When applied to the Late Cretaceous marine dataset, results suggest that GARs for epicontinental seas generally have higher slopes than their open-ocean counterparts, which suggests a greater degree of compositional heterogeneity as a function of geographic area. These differences are consistent with the emerging understanding of physical differences in the nature of water masses between the two settings. To further broaden and confirm these findings, however, it will be important to expand both the geographic and stratigraphic purview of future analyses, to include more open-ocean settings in particular (e.g., the Pacific coasts of North America and Japan), and other stratigraphic intervals during the Mesozoic, for which substantial data are available for both settings.
